# The ratio of means method as an alternative to mean differences for analyzing continuous outcome variables in meta-analysis: A simulation study

**DOI:** 10.1186/1471-2288-8-32

**Published:** 2008-05-21

**Authors:** Jan O Friedrich, Neill KJ Adhikari, Joseph Beyene

**Affiliations:** 1 Department of Medicine, University of Toronto, Toronto, Canada; 2 Interdepartmental Division of Critical Care, University of Toronto, Toronto, Canada; 3 Critical Care and Medicine Departments and Li Ka Shing Knowledge Institute, St. Michael's Hospital, Toronto, Canada; 4 Department of Critical Care Medicine and Sunnybrook Research Institute, Sunnybrook Health Sciences Centre, Toronto, Canada; 5 Department of Public Health Sciences, University of Toronto, Toronto, Canada; 6 Child Health Evaluative Sciences, Hospital for Sick Children Research Institute, Toronto, Canada

## Abstract

**Background:**

Meta-analysis of continuous outcomes traditionally uses mean difference (MD) or standardized mean difference (SMD; mean difference in pooled standard deviation (SD) units). We recently used an alternative ratio of mean values (RoM) method, calculating RoM for each study and estimating its variance by the delta method. SMD and RoM allow pooling of outcomes expressed in different units and comparisons of effect sizes across interventions, but RoM interpretation does not require knowledge of the pooled SD, a quantity generally unknown to clinicians.

**Objectives and methods:**

To evaluate performance characteristics of MD, SMD and RoM using simulated data sets and representative parameters.

**Results:**

MD was relatively bias-free. SMD exhibited bias (~5%) towards no effect in scenarios with few patients per trial (n = 10). RoM was bias-free except for some scenarios with broad distributions (SD 70% of mean value) and medium-to-large effect sizes (0.5–0.8 pooled SD units), for which bias ranged from -4 to 2% (negative sign denotes bias towards no effect). Coverage was as expected for all effect measures in all scenarios with minimal bias. RoM scenarios with bias towards no effect exceeding 1.5% demonstrated lower coverage of the 95% confidence interval than MD (89–92% vs. 92–94%). Statistical power was similar. Compared to MD, simulated heterogeneity estimates for SMD and RoM were lower in scenarios with bias because of decreased weighting of extreme values. Otherwise, heterogeneity was similar among methods.

**Conclusion:**

Simulation suggests that RoM exhibits comparable performance characteristics to MD and SMD. Favourable statistical properties and potentially simplified clinical interpretation justify the ratio of means method as an option for pooling continuous outcomes.

## Background

Meta-analysis is a method of statistically combining results of similar studies [1]. For binary outcome variables both difference and ratio methods are commonly used. For each study, the risk difference is the difference in proportions of patients experiencing the outcome of interest between the experimental and control groups, the risk ratio is the ratio of these proportions, and the odds ratio is the ratio of the odds. Meta-analytic techniques are used to combine each study's effect measure to generate a pooled effect measure. Standard meta-analytic procedures for each of these effect measures also estimate heterogeneity, which is the variability in treatment effects of individual trials beyond that expected by chance. Each effect measure (risk difference, risk ratio, odds ratio) has advantages and disadvantages in terms of consistency, mathematical properties, and ease of interpretation, implying that none is universally optimal [2].

In contrast, for continuous outcome variables, only difference methods are commonly used for group comparison studies [3]. If the outcome of interest is measured in identical units across trials, then the effect measure for each trial is the difference in means, and the pooled effect measure is the mean difference (MD), which more accurately should be described as the weighted mean of mean differences. If the outcome of interest is measured in different units, then each trial's effect measure is the difference in mean values divided by the pooled standard deviation of the two groups, and the pooled effect measure is the standardized mean difference (SMD), which more accurately should be described as the weighted mean of standardized mean differences. Normalizing the differences using the standard deviation allows pooling of such results, in addition to allowing comparison of effect sizes across unrelated interventions. By convention [4], SMD's of 0.2, 0.5, and 0.8 are considered "small", "medium", and "large" effect sizes, respectively. When trials in meta-analyses are weighted by the inverse of the variance of the effect measure (the weighting scheme generally used for MD and SMD), the pooled SMD has the unfavorable statistical property of negative bias (i.e. towards the null value) [5,6]. Alternative methods of estimating the variance of individual trial SMDs used in the inverse variance method have been proposed to minimize this bias [5,6].

In principle, meta-analysts could also use ratio methods to analyze continuous outcomes, by calculating a ratio of mean values instead of a difference. Since the ratio is unitless, this calculation can be carried out regardless of the specific units used in individual trials. Moreover, as with SMD, a ratio can be used to combine related but different outcomes (e.g. quality of life scales). We have recently used this Ratio of Means (RoM) method in meta-analyses [7-9] in which we estimated the variance of this ratio using the delta method [10]. For this method, each individual study RoM is converted to its natural logarithm before being pooled, and the pooled result is then back transformed, similar to odds and risk ratio calculations used for binary outcomes. Table 1 presents the pooled results for the continuous variables from the meta-analysis of low-dose dopamine for renal dysfunction [7], analyzed using mean difference methods and the RoM method, in addition to heterogeneity expressed using the *I*^2 ^measure. (*I*^2 ^is the percentage of total variation in results across studies due to heterogeneity rather than chance [11,12].) Table 1 shows similar results among the three methods. The point estimates are similar in direction (i.e. a positive mean difference or standardized mean difference corresponds to a RoM greater than one, while a negative mean difference corresponds to a RoM less than one). The confidence intervals result in similar p-values for statistically significant increases or decreases for each of these parameters. Finally, heterogeneity is similar.

**Table 1:** Renal Physiological Parameters from Low-Dose Dopamine Meta-Analysis 1 Day After Starting Therapy [7].

				Effect Measure		
	
Parameter	Number of Trials	Number of Patients		MD	SMD	RoM
						
Urine Output	33	1654	Estimate	--	0.49	1.24
			95% CI	--	0.29 to 0.69	1.14 to 1.35
			p-value	--	<0.001	<0.001
			*I*^2^	--	71%	77%
Serum Creatinine	32	1807	Estimate	-3.51	-0.28	0.96
			95% CI	-6.71 to -0.23	-0.51 to -0.06	0.93 to 0.99
			p-value	0.04	0.01	0.01
			*I*^2^	73%	79%	73%
Creatinine Clearance	22	1077	Estimate	--	0.10	1.06
			95% CI	--	-0.02 to 0.22	1.01 to 1.11
			p-value	--	0.10	0.02
			*I*^2^	--	0%	0%

The pooled effect measure results are presented along with their 95% confidence intervals and null hypothesis p-values for each of the three renal physiological variables evaluated in the meta-analysis, urine output, serum creatinine, and creatinine clearance, 1 day after the start of therapy. The degree of heterogeneity, expressed using the *I*^2 ^statistic for each of the pooled effect measures for each of the variables is also shown. For urine output and creatinine clearance, MD could not be used because units differed across studies. In contrast, all serum creatinine values were expressed as or could be converted to identical units (μmol/L), allowing this variable to also be analyzed using MD.

Abbreviations: CI – confidence interval, *I*^2 ^– *I*^2 ^heterogeneity statistic, MD – mean difference, RoM – ratio of means, SMD – standardized mean difference

Given the similarity of these results, the objective of this current study was to test the hypothesis that MD, SMD, and RoM methods exhibit comparable performance characteristics in terms of bias, coverage and statistical power, using simulated data sets with a range of parameters commonly encountered in meta-analyses.

## Methods

### The RoM Effect Measure

For mean difference meta-analysis, one calculates a difference in mean values between the experimental and control groups for each study. (A review of the inverse-variance weighted fixed and random effects models and calculation of the point estimates and variances for MD and SMD using standard methods [including a correction factor for small samples for SMD], can be found in the Appendix). Instead of calculating a difference in mean values between the experimental and control groups, one can calculate a ratio of mean values. The following uses the natural logarithm scale to carry out such calculations, similar to statistical procedures for binary effect measures (risk ratio and odds ratio), due to its desirable statistical properties [14].

For a study reporting a continuous outcome, let the mean, standard deviation, and number of patients be denoted by *mean*_*exp*_, *sd*_*exp*_, and *n*_*exp*_respectively in the experimental group and *mean*_*contr*_, *sd*_*contr*_, and *n*_*contr*_, respectively in the control group. The $\text{RoM}=\frac{mean_{\exp}}{mean_{contr}}$ and the variance (*Var*) of its natural logarithm is estimated as follows:

Var[ln⁡(meanexp⁡meancontr)]=Var[ln⁡(meanexp⁡)−ln⁡(meancontr)]=Var[ln⁡(meanexp⁡)]+Var[ln⁡(meancontr)]     [since the groups are independent]=(1meanexp⁡)2Var(meanexp⁡)+(1meancontr)2Var(meancontr)=1nexp⁡(sdexp⁡meanexp⁡)2+1ncontr(sdcontrmeancontr)2[since for random variable X, Var(meanX)=Var(X)nX=sdX2nX]

The natural logarithm transformed ratios are aggregated across studies using the generalized inverse variance method described in the Appendix. The pooled transformed ratio is then back transformed to obtain a pooled ratio and 95% confidence interval (CI), as follows:

$$95\%CI=\exp\left\{\left[\ln\left(\frac{mean_{\exp}}{mean_{contr}}\right)\right]\pm 1.96\sqrt{Var\left[\ln\left(\frac{mean_{\exp}}{mean_{contr}}\right)\right]}\right\}$$

Log transformation of the ratio of mean values, a non-normally distributed function, allows this approximation of the 95% confidence interval of this approximately normally distributed transformed function. This approach is similar to that applied to other ratio methods such as OR and RR, used for binary group comparison studies.

As the ratio of means method is unitless, this method can be used irrespective of the units used in trial outcome measures. Using the delta method limited to first order terms results in a straightforward formula to estimate the variance of the ratio. Second order terms would be raised to the fourth power and are not included as they would not increase the variance by much. For example, even choosing simulation parameters that maximized the contribution of these second order terms (ratio of the standard deviation to the mean equal to 0.7, and n = 10 patients per trial arm [see below]), would increase the variance estimate by less than 2.5%.

### Design of the Simulation Study

The parameters and their assigned values used to simulate continuous variable meta-analysis data sets for the individual scenarios are shown in Table 2. The values were chosen to be representative of typical meta-analyses exhibiting a range of standard deviations, number of trials, number of participants per trial, effect sizes, and heterogeneity. The standard deviation of the control group, expressed as a percentage of the mean value in the control group, was varied between 10% and 70% to reflect a narrow to broad distribution around the mean value. The standard deviations of the control and experimental groups were assumed to be equal for all simulations. The number of trials (k) ranged from 5 to 30. The number of patients per trial arm (n) was set to either 10 or 100 in each of the experimental and control groups, and all trials within each simulation were assumed to have the same number of patients. The effect size, expressed in pooled standard deviation units (i.e. SMD), was set at 0.2, 0.5, and 0.8 [4].

**Table 2:** Parameter Values Used in the Simulated Data Sets

Varied Parameter	Assigned Values
Standard Deviation (percentage of control mean value)	10%, 40%, 70%
Number of Trials	5, 10, 30
Number of Experimental and Control Patients Per Trial Arm	10, 100
Effect Size (in standard deviation units)	0.2, 0.5, 0.8
Heterogeneity of Mean Values (in standard deviation units)	0, 0.5

For each simulated scenario, k simulated study means and standard deviations were calculated from a collection of n individual values randomly sampled from a normal distribution. This was done independently for the control and experimental groups. For the control group the normal distribution from which values were randomly sampled had a mean value set to 100, resulting in a standard deviation of 10, 40, or 70. For the experimental group the normal distribution from which values were randomly sampled had a mean value of [100 + (effect size) × (standard deviation)] and the same standard deviation as the control group. Using the simulated study mean values and standard deviations, meta-analysis was carried out using MD, SMD, and RoM, with inverse variance weighting and a random effects model as described in the Appendix. With the parameters described above, the expected MD = (effect size) × (standard deviation), the expected SMD = effect size, and the expected RoM = 1 + [(effect size) × (standard deviation)/(mean value in control group[= 100])], where effect size varies as 0.2, 0.5 and 0.8, and the standard deviation varies as 10, 40, and 70.

Heterogeneity for each scenario was introduced by setting *τ *= 0.5 standard deviation units. This was achieved by introducing an additional study-specific standard deviation equal to 0.5/√2 standard deviation units to both the experimental and the control groups, since the study-specific standard deviation of the difference between experimental and control groups is given by √[(0.5/√2)^2 ^+ (0.5/√2)^2^] = 0.5 standard deviation units. In other words, study-specific variance was added to experimental and control group means but the baseline difference and ratio in mean values was held constant. Since a given degree of result heterogeneity may be reflected differently in the difference methods (MD and SMD) compared to the ratio method (RoM), heterogeneity was added at the level of the individual mean values rather than the level of the treatment effects to ensure that the degree of heterogeneity added was comparable between the three methods. Heterogeneity of each meta-analysis scenario is presented using *I*^2^. Since *I*^2 ^= *τ*^2^/(*τ*^2 ^+ s^2^), where s^2 ^is the variance of the effect measure, as described in the Appendix, the expected value for *I*^2 ^for *τ *= 0.5 can be calculated to be 56% when n = 10 patients per trial arm, corresponding to the introduction of a moderate (i.e. *I*^2 ^= 50–75%) degree of heterogeneity, and 93% when n = 100 patients per trial arm, corresponding to a high (i.e. *I*^2 ^> 75%) degree of heterogeneity [11,12].

The baseline scenarios assumed equal numbers of participants in both the experimental and control arms and were constructed by randomly selecting data points from normally distributed data. Separate sensitivity analyses were also carried out to determine 1) the effect of unequal numbers of participants (chosen to have a 2:1 and 1:2 experimental:control arm ratio but keeping the total number of participants constant (i.e. 14:6 instead of 10:10 and 134:66 instead of 100:100)) and 2) the effect of selecting the data points from an underlying skewed distribution. The skewed distribution was empirically constructed by mixing a combination of 3 normal distributions with identical standard deviations (0.24) centered at 0.84, 1.42 and 1.92 and weighted 77%, 17%, and 6% respectively in the overall mixed skewed distribution. This created a graphical distribution appearing markedly skewed on visual inspection with an overall mean of unity and overall standard deviation similar to that of the middle normally distributed data scenario (i.e. 40% of the control mean value), but skewness (third standardized moment about the mean [15]) of 0.88.

For each scenario, data points were generated and analyzed 10,000 times and performance characteristics of each effect measure were assessed. These consisted of bias (expressed as a percentage of the true parameter value, directed away or towards the null value [zero for MD and SMD, and one for RoM]), coverage (of the 95% confidence interval of the simulated result, i.e. the percentage of time that the true parameter value falls within the 95% confidence interval of the simulated result), statistical power (the percentage of time that the 95% confidence interval of the simulated result yields a significant treatment effect, by excluding zero for MD and SMD or one for RoM), and heterogeneity (expressed as *I*^2^). Simulations were programmed and carried out using SAS (version 8.2, Cary, NC).

## Results

Table 3 presents simulation results for the baseline scenarios (standard deviation 40% of the control mean, equal numbers of patients in the control and experimental groups, underlying normal distribution of the individual data points), for each combination of effect size (0.2, 0.5, and 0.8 standard deviation units), number of patients (10 and 100 control and experimental patients per trial), and number of trials (5, 10, and 30). Results for similar scenarios except with lower (10% of the control mean) or higher (70% of the control mean) standard deviations are shown in Tables 4 and 5. The skewed distribution results were similar (Table 6).

**Table 3:** Simulation Results (Normal Distribution, Equal Experimental and Control Groups, Standard Deviation 40% of Control Mean Value).

			% Bias						% Coverage						% Statistical Power						*I*^2^(%)		
						
			*τ *= 0s			*τ *= 0.5s			*τ *= 0s			*τ *= 0.5s			*τ *= 0s			*τ *0.5s			*τ *0.5s		
							
Δ	n (exp/contr)	k	MD	SMD	RoM	MD	SMD	RoM	MD	SMD	RoM	MD	SMD	RoM	MD	SMD	RoM	MD	SMD	RoM	MD	SMD	RoM
SMD = 0.2	10/10	5	0	-4	0	0	-5	1	95	97	95	90	92	90	15	11	14	15	13	14	59	48	55
		10	0	-5	0	1	-5	0	95	97	95	92	93	92	27	22	26	19	18	19	60	48	56
		30	0	-5	0	1	-6	0	94	96	95	94	94	94	66	61	64	39	38	38	60	48	56
	
MD = 8	100/100	5	0	0	0	0	0	1	96	97	96	88	88	87	82	82	82	21	21	21	93	92	92
RoM = 1.08		10	0	0	0	0	0	0	96	96	96	92	92	91	99	99	99	27	27	28	93	92	92
		30	0	0	0	0	0	0	96	96	96	94	94	94	100	100	100	56	58	59	93	92	92

																							

SMD = 0.5	10/10	5	0	-4	0	0	-5	0	95	97	95	90	92	90	64	57	62	43	40	42	59	48	56
		10	0	-5	0	0	-5	0	95	97	95	92	93	92	91	89	90	66	64	65	60	48	56
		30	0	-5	0	0	-6	0	94	96	94	94	93	93	100	100	100	98	97	97	60	47	57
	
MD = 20	100/100	5	0	0	0	0	0	1	96	97	96	88	88	88	100	100	100	61	61	61	93	92	92
RoM = 1.2		10	0	0	0	0	0	0	96	96	96	92	92	91	100	100	100	85	86	86	93	92	92
		30	0	0	0	0	-1	0	96	96	96	94	94	93	100	100	100	100	100	100	93	92	92

																							

SMD = 0.8	10/10	5	0	-4	0	0	-5	0	95	97	95	90	92	90	95	94	95	75	72	73	59	47	56
		10	0	-5	0	0	-5	0	95	96	95	92	92	92	100	100	100	95	95	95	60	47	57
		30	0	-5	-1	0	-6	0	94	94	94	94	92	93	100	100	100	100	100	100	60	46	57
	
MD = 32	100/100	5	0	0	0	0	0	1	96	96	96	88	88	87	100	100	100	91	91	91	93	92	92
RoM = 1.32		10	0	0	0	0	0	1	96	96	96	92	92	91	100	100	100	100	100	100	93	91	92
		30	0	0	0	0	-1	0	96	96	96	94	94	93	100	100	100	100	100	100	93	91	92

Results of 10,000 simulations per scenario with a standard deviation equal to 40% of the control mean, for each combination of effect size (0.2, 0.5, and 0.8 standard deviation units), number of patients (10/10 and 100/100 experimental/control patients per trial), and number of trials (5, 10, and 30). The "% Bias" columns show the bias of each effect measure (MD, SMD, RoM) expressed as percentages of the expected values (negative sign denotes less than expected value), with and without heterogeneity. The "% coverage" columns show the percentage of cases that the true value falls within the 95% confidence interval of the simulated result, with and without heterogeneity. The "% statistical power" columns show the percentage of cases that the 95% confidence interval of the simulated result yields a significant treatment effect (i.e. excluding zero for MD and SMD, and one for RoM), with and without heterogeneity. The *I*^2^ column shows the degree of heterogeneity only for the scenarios in which heterogeneity was introduced. (For all the scenarios without heterogeneity, the ratio of Q/(k-1) was close to unity as expected, corresponding to *I*^2 ^= 0 [data not shown].)

Abbreviations for Table and Legend: contr – control, exp – experimental, *I*^2 ^– *I*^2 ^heterogeneity measure, k – number of trials in each meta-analysis, n – number of experimental or number of control patients per trial, Q – Cochran's Q statistic for heterogeneity, MD – mean difference, RoM – ratio of means, s – standard deviation units, SMD – standardized mean difference.

**Table 4:** Simulation Results (Normal Distribution, Equal Experimental and Control Groups, Standard Deviation 10% of Control Mean Value).

			% Bias						% Coverage						% Statistical Power						*I*^2^(%)		
						
			*τ *= 0s			*τ *= 0.5s			*τ *= 0s			*τ *= 0.5s			*τ *= 0s			*τ *0.5s			*τ *0.5s		
							
Δ	n (exp/contr)	k	MD	SMD	RoM	MD	SMD	RoM	MD	SMD	RoM	MD	SMD	RoM	MD	SMD	RoM	MD	SMD	RoM	MD	SMD	RoM
SMD = 0.2	10/10	5	0	-4	0	0	-5	0	95	97	95	90	92	90	15	11	15	15	13	15	59	48	59
		10	0	-5	0	1	-5	0	95	97	95	92	93	92	27	22	27	19	18	19	60	48	60
		30	0	-5	0	1	-6	0	94	96	94	94	94	94	66	61	66	39	38	39	60	48	60
	
MD = 2	100/100	5	0	0	0	0	0	0	96	97	96	88	88	88	82	82	82	21	21	21	93	92	93
RoM = 1.02		10	0	0	0	0	0	0	96	96	96	92	92	92	99	99	99	27	27	27	93	92	93
		30	0	0	0	0	0	0	96	96	96	94	94	94	100	100	100	56	58	57	93	92	93

																							

SMD = 0.5	10/10	5	0	-4	0	0	-5	0	95	97	95	90	92	90	64	57	63	43	40	43	59	48	59
		10	0	-5	0	0	-5	0	95	97	95	92	93	92	91	89	91	66	64	66	60	48	60
		30	0	-5	0	0	-6	0	94	96	94	94	93	94	100	100	100	98	97	98	60	47	60
	
MD = 5	100/100	5	0	0	0	0	0	0	96	97	96	88	88	88	100	100	100	61	61	61	93	92	93
RoM = 1.05		10	0	0	0	0	0	0	96	96	96	92	92	91	100	100	100	85	86	85	93	92	93
		30	0	0	0	0	-1	0	96	96	96	94	94	94	100	100	100	100	100	100	93	92	93

																							

SMD = 0.8	10/10	5	0	-4	0	0	-5	0	95	97	95	90	92	90	95	94	95	75	72	74	59	47	59
		10	0	-5	0	0	-5	0	95	96	95	92	92	92	100	100	100	95	95	95	60	47	60
		30	0	-5	0	0	-6	0	94	94	94	94	92	94	100	100	100	100	100	100	60	46	60
	
MD = 8	100/100	5	0	0	0	0	0	0	96	96	96	88	88	88	100	100	100	91	91	91	93	92	93
RoM = 1.08		10	0	0	0	0	0	0	96	96	96	92	92	92	100	100	100	100	100	100	93	91	93
		30	0	0	0	0	-1	0	96	96	96	94	94	94	100	100	100	100	100	100	93	91	93

Results of 10,000 simulations per scenario with a standard deviation equal to 10% of the control mean, for each combination of effect size (0.2, 0.5, and 0.8 standard deviation units), number of patients (10/10 and 100/100 experimental/control patients per trial), and number of trials (5, 10, and 30). The "% Bias" columns show the bias of each effect measure (MD, SMD, RoM) expressed as percentages of the expected values (negative sign denotes less than expected value), with and without heterogeneity. The "% coverage" columns show the percentage of cases that the true value falls within the 95% confidence interval of the simulated result, with and without heterogeneity. The "% statistical power" columns show the percentage of cases that the 95% confidence interval of the simulated result yields a significant treatment effect (i.e. excluding zero for MD and SMD, and one for RoM), with and without heterogeneity. The *I*^2^ column shows the degree of heterogeneity only for the scenarios in which heterogeneity was introduced. (For all the scenarios without heterogeneity, the ratio of Q/(k-1) was close to unity as expected, corresponding to *I*^2 ^= 0 [data not shown].)

Abbreviations for Table and Legend: contr – control, exp – experimental, *I*^2 ^– *I*^2 ^heterogeneity measure, k – number of trials in each meta-analysis, n – number of experimental or number of control patients per trial, Q – Cochran's Q statistic for heterogeneity, MD – mean difference, RoM – ratio of means, s – standard deviation units, SMD – standardized mean difference.

**Table 5:** Simulation Results (Normal Distribution, Equal Experimental and Control Groups, Standard Deviation 70% of Control Mean Value).

			% Bias						% Coverage						% Statistical Power						*I*^2^(%)		
						
			*τ *= 0s			*τ *= 0.5s			*τ *= 0s			*τ *= 0.5s			*τ *= 0s			*τ *0.5s			*τ *0.5s		
							
Δ	n (exp/contr)	k	MD	SMD	RoM	MD	SMD	RoM	MD	SMD	RoM	MD	SMD	RoM	MD	SMD	RoM	MD	SMD	RoM	MD	SMD	RoM
SMD = 0.2	10/10	5	0	-4	0	-1	-5	0	95	97	96	90	92	91	15	11	12	15	13	12	59	48	45
		10	0	-5	-1	0	-5	-1	95	97	96	92	93	93	27	22	23	19	18	16	60	48	45
		30	0	-5	-1	0	-6	-1	94	96	95	94	94	93	66	61	60	38	38	34	60	47	46
	
MD = 14	100/100	5	0	0	0	0	-1	2	96	97	96	88	88	87	82	82	82	21	21	22	93	92	91
RoM = 1.14		10	0	0	0	0	0	1	96	96	96	92	92	90	99	99	99	27	27	31	93	92	91
		30	0	0	0	0	0	1	96	96	96	94	94	92	100	100	100	56	58	62	93	92	91

																							

SMD = 0.5	10/10	5	0	-4	-1	0	-5	-1	95	97	95	90	92	91	64	57	58	43	40	38	59	48	47
		10	0	-5	-2	0	-5	-2	95	97	95	92	93	92	91	89	88	66	64	61	60	47	47
		30	0	-5	-2	0	-6	-3	94	96	92	94	93	91	100	100	100	98	97	97	60	47	48
	
MD = 35	100/100	5	0	0	0	0	0	2	96	97	96	88	88	87	100	100	100	61	61	63	93	92	91
RoM = 1.35		10	0	0	0	0	0	1	96	96	96	92	92	90	100	100	100	85	86	88	93	92	91
		30	0	0	0	0	-1	1	96	96	95	94	94	92	100	100	100	100	100	100	93	92	91

																							

SMD = 0.8	10/10	5	0	-4	-1	0	-5	-2	95	97	95	90	92	90	95	94	93	74	72	71	59	47	48
		10	0	-5	-2	0	-5	-3	95	96	94	92	92	91	100	100	100	95	95	94	60	46	48
		30	0	-5	-3	0	-6	-4	94	94	90	94	92	89	100	100	100	100	100	100	60	46	49
	
MD = 56	100/100	5	0	0	0	0	0	2	96	96	96	88	88	87	100	100	100	91	91	92	93	92	91
RoM= 1.56		10	0	0	0	0	0	2	96	96	96	92	92	90	100	100	100	100	100	100	93	91	91
		30	0	0	0	0	-1	1	96	96	95	94	94	92	100	100	100	100	100	100	93	91	91

Results of 10,000 simulations per scenario with a standard deviation equal to 70% of the control mean, for each combination of effect size (0.2, 0.5, and 0.8 standard deviation units), number of patients (10/10 and 100/100 experimental/control patients per trial), and number of trials (5, 10, and 30). The "% Bias" columns show the bias of each effect measure (MD, SMD, RoM) expressed as percentages of the expected values (negative sign denotes less than expected value), with and without heterogeneity. The "% coverage" columns show the percentage of cases that the true value falls within the 95% confidence interval of the simulated result, with and without heterogeneity. The "% statistical power" columns show the percentage of cases that the 95% confidence interval of the simulated result yields a significant treatment effect (i.e. excluding zero for MD and SMD, and one for RoM), with and without heterogeneity. The *I*^2^ column shows the degree of heterogeneity only for the scenarios in which heterogeneity was introduced. (For all the scenarios without heterogeneity, the ratio of Q/(k-1) was close to unity as expected, corresponding to *I*^2 ^= 0 [data not shown].)

Abbreviations for Table and Legend: contr – control, exp – experimental, *I*^2 ^– *I*^2 ^heterogeneity measure, k – number of trials in each meta-analysis, n – number of experimental or number of control patients per trial, Q – Cochran's Q statistic for heterogeneity, MD – mean difference, RoM – ratio of means, s – standard deviation units, SMD – standardized mean difference.

**Table 6:** Simulation Results (Skewed Distribution, Equal Experimental and Control Groups, Standard Deviation 40% of Control Mean Value).

			% Bias						% Coverage						% Statistical Power						*I*^2^(%)		
						
			*τ *= 0s			*τ *= 0.5s			*τ *= 0s			*τ *= 0.5s			*τ *= 0s			*τ *0.5s			*τ *0.5s		
							
Δ	N (exp/contr)	k	MD	SMD	RoM	MD	SMD	RoM	MD	SMD	RoM	MD	SMD	RoM	MD	SMD	RoM	MD	SMD	RoM	MD	SMD	RoM
SMD = 0.2	10/10	5	0	-2	0	-1	-5	1	95	97	95	89	92	90	16	12	16	15	13	15	60	50	60
		10	0	-3	0	0	-4	0	94	97	94	92	93	92	29	22	28	19	17	19	61	49	61
		30	0	-4	0	0	-5	0	94	96	94	94	94	94	68	62	67	38	37	36	61	49	61
	
MD = 8	100/100	5	0	0	0	1	0	1	96	96	96	88	88	88	82	81	82	21	21	21	93	92	92
RoM = 1.08		10	0	-1	0	0	0	0	96	96	96	92	92	91	99	99	99	27	28	28	93	92	92
		30	0	0	0	0	0	0	96	96	96	94	93	93	100	100	100	56	58	59	93	92	92

																							

SMD = 0.5	10/10	5	0	-2	1	-1	-4	1	95	97	95	89	92	90	65	59	64	43	40	42	60	49	60
		10	0	-3	0	0	-4	0	94	97	94	92	93	92	92	90	91	65	63	63	61	49	61
		30	0	-4	0	0	-5	0	94	96	94	94	94	94	100	100	100	98	98	98	61	48	61
	
MD = 20	100/100	5	0	0	0	0	0	1	96	96	96	88	88	88	100	100	100	61	61	61	93	92	92
RoM = 1.2		10	0	0	0	0	0	0	96	96	96	92	91	91	100	100	100	85	86	86	93	92	92
		30	0	0	0	0	0	0	96	96	96	94	93	93	100	100	100	100	100	100	93	92	92

																							

SMD = 0.8	10/10	5	0	-2	1	0	-3	1	95	97	95	89	92	90	95	94	95	74	72	73	60	48	60
		10	0	-3	1	0	-4	0	94	96	94	92	92	92	100	100	100	95	95	95	61	48	61
		30	0	-4	0	0	-5	0	94	95	94	94	93	94	100	100	100	100	100	100	61	48	62
	
MD = 32	100/100	5	0	0	0	0	0	1	96	96	96	88	88	88	100	100	100	91	91	91	93	92	92
RoM = 1.32		10	0	0	0	0	0	1	96	96	96	92	92	91	100	100	100	100	100	100	93	92	92
		30	0	0	0	0	0	0	96	96	96	94	93	93	100	100	100	100	100	100	93	91	92

Results of 10,000 simulations per scenario with a standard deviation equal to 40% of the control mean, for each combination of effect size (0.2, 0.5, and 0.8 standard deviation units), number of patients (10/10 and 100/100 experimental/control patients per trial), and number of trials (5, 10, and 30) assuming a skewed distribution described in the Methods. The "% Bias" columns show the bias of each effect measure (MD, SMD, RoM) expressed as percentages of the expected values (negative sign denotes less than expected value), with and without heterogeneity. The "% coverage" columns show the percentage of cases that the true value falls within the 95% confidence interval of the simulated result, with and without heterogeneity. The "% statistical power" columns show the percentage of cases that the 95% confidence interval of the simulated result yields a significant treatment effect (i.e. excluding zero for MD and SMD, and one for RoM), with and without heterogeneity. The *I*^2^ column shows the degree of heterogeneity only for the scenarios in which heterogeneity was introduced. (For all the scenarios without heterogeneity, the ratio of Q/(k-1) was close to unity as expected, corresponding to *I*^2 ^= 0 [data not shown].)

Abbreviations for Table and Legend: contr – control, exp – experimental, *I*^2 ^– *I*^2 ^heterogeneity measure, k – number of trials in each meta-analysis, n – number of experimental or number of control patients per trial, Q – Cochran's Q statistic for heterogeneity, MD – mean difference, RoM – ratio of means, s – standard deviation units, SMD – standardized mean difference.

### Bias

The MD method exhibits minimal bias (less than 0.5%) in almost all of scenarios. In contrast, there is one principal source of bias for the SMD method and two for the RoM method.

#### SMD Bias Towards No Effect with Smaller Trials

SMD is biased towards zero or no effect, with the bias more prominent when the number of patients per study is small. Table 3 shows this bias to be (-)4 to 6% in the baseline scenario with 10 patients per trial, regardless of the number of trials. The bias decreases in the 100-patient per trial scenarios. The lower weighting of extreme values (i.e. values far from no effect [zero]) in the SMD method also results in decreased heterogeneity (*I*^2^), in the scenarios with 10 patients per trial where the bias is largest (discussed in the heterogeneity section below). Sampling variance alone results in bias toward zero, but this bias is even larger when heterogeneity is present since this results in a further increase in dispersion (or the effective variance) of the results. These findings are consistent with theoretical considerations (see Appendix).

#### RoM Bias

In contrast, the RoM bias depends on the relative effects of two competing sources of bias. The first is a negative bias towards unity or no effect due to properties of the variance of ln(RoM) and is most pronounced when the number of patients per trial is small. The second is a bias away from unity or no effect occurring when heterogeneity is present, due to properties of RoM. Although bias from both sources is absent or less than 0.5% in all scenarios with 100 patients per trial and no heterogeneity, one or both sources of bias can be significant in other scenarios. These are described in more detail below.

##### RoM Bias Towards No Effect with Smaller Trials

To understand the bias towards unity or no effect, one must consider the factors influencing the variance of ln(RoM) described in the Methods. As in the scenarios studied with equal standard deviations in the control and experimental groups, consider RoM >1, where the experimental mean is greater than the control mean. In this situation the contribution of the experimental group's relative error to the variance of ln(RoM) is smaller than that of the control group's relative error. As RoM increases, either the experimental mean value (*mean*_*exp*_) increases for a given control mean value (*mean*_*contr*_) or *mean*_*contr *_falls for a given *mean*_*exp*_. In the former case, the term (1/*mean*_*exp*_)^2 ^falls and the variance of ln(RoM) becomes relatively smaller, compared to lower RoM values. In the latter case, the term (1/*mean*_*contr*_)^2 ^increases and the variance of ln(RoM) becomes relatively larger, compared to lower RoM values. Because of the different relative error term contributions discussed above, the decrease in the experimental group relative error term determined by (1/*mean*_*exp*_)^2 ^is smaller than the increase in the control group relative error term determined by (1/*mean*_*contr*_)^2^. Thus, when these effects are averaged, the overall effect is that higher RoM values have a higher variance and therefore receive relatively lower weighting in the inverse variance weighted meta-analysis, leading to bias towards unity or no effect. This bias is accentuated by i) larger standard deviations, ii) higher heterogeneity (due to larger effective standard deviations), and iii) smaller trials. The bias is best demonstrated in the scenarios without heterogeneity in which the standard deviation is 70% of the mean control value, the number of patients per trial is 10 and the effect size is moderate to large, as shown in Table 5. (This bias is also present in the scenarios with heterogeneity and 10 patients per trial shown in Table 5; however, the overall bias in these scenarios is due to the combined effect of both this bias towards unity discussed in this section and a bias away from unity discussed in the next section.) Under these conditions, the magnitude of this bias ranges up to 2–3%. Due to its inverse dependence on study size it decreases to less than 0.5% in the scenarios without heterogeneity enrolling 100 patients per trial. The bias is also accentuated in scenarios with either larger RoM or a ratio of the number of patients in the experimental group to the number of patients in the control group (*n*_*exp*_/*n*_*contr*_) >1. This occurs because as either *mean*_*exp *_increases relative to *mean*_*contr *_or *n*_*exp *_increases relative to *n*_*contr*_, the term (1/*n*_*exp*_*mean*_*exp*_^2^) decreases and changes in the control group relative error predominate to an even greater extent. For example, compare the results from scenarios without heterogeneity shown in Table 3 with a 1:1 experimental to control patient ratio to those shown in Table 7 with a 2:1 experimental to control patient ratio. In contrast, increasing *n*_*cont *_relative to *n*_*exp *_decreases the contribution of the control group's relative error. This decreases the magnitude of the bias towards unity and can even change the direction of the bias from negative to positive (i.e. to a bias away from unity or no effect) if the ratio *n*_*contr*_/*n*_*exp *_is increased to a value greater than *mean*_*exp*_^2^/*mean*_*contr*_^2 ^(RoM^2^). This is illustrated in Table 8 where *n*_*contr*_/*n*_*exp *_= 2 and RoM^2 ^< 2.

**Table 7:** Simulation Results (Normal Distribution, 2:1 Experimental to Control Group Sizes, Standard Deviation 40% of Control Mean Value).

			% Bias						% Coverage						% Statistical Power						*I*^2^(%)		
						
			*τ *= 0s			*τ *= 0.5s			*τ *= 0s			*τ *= 0.5s			*τ *= 0s			*τ *0.5s			*τ *0.5s		
							
Δ	n (exp/contr)	k	MD	SMD	RoM	MD	SMD	RoM	MD	SMD	RoM	MD	SMD	RoM	MD	SMD	RoM	MD	SMD	RoM	MD	SMD	RoM
SMD = 0.2	14/6	5	-1	-5	0	0	-5	0	94	97	94	90	92	90	15	10	11	14	12	12	59	44	54
		10	1	-4	-1	2	-4	-1	94	97	93	92	93	91	25	18	18	18	16	14	60	44	55
		30	0	-5	-1	1	-6	-1	94	97	91	93	94	93	59	53	44	36	35	27	60	44	55
	
MD = 8	134/66	5	0	0	0	0	-1	0	96	97	97	88	88	88	78	77	76	21	21	21	92	91	91
RoM = 1.08		10	0	0	0	0	-1	0	96	96	96	92	91	91	98	98	97	27	27	27	92	91	91
		30	0	0	0	0	-1	0	96	96	96	94	94	94	100	100	100	56	57	57	92	91	91

																							

SMD = 0.5	14/6	5	0	-4	-1	0	-5	0	94	97	94	90	92	90	57	50	50	41	37	36	59	44	54
		10	0	-5	-1	1	-5	-1	94	97	93	92	93	91	86	83	79	62	61	56	60	44	55
		30	0	-5	-1	0	-6	-1	94	96	91	93	93	92	100	100	100	96	97	94	60	43	56
	
MD = 20	134/66	5	0	0	0	0	-1	1	96	96	97	88	88	88	100	100	100	60	61	60	92	91	91
RoM = 1.2		10	0	0	0	0	-1	0	96	96	96	92	92	91	100	100	100	85	86	85	92	91	91
		30	0	0	0	0	-1	0	96	96	95	94	94	94	100	100	100	100	100	100	92	91	91

																							

SMD = 0.8	14/6	5	0	-4	-1	0	-5	0	94	97	93	90	92	90	91	89	87	71	69	66	59	43	54
		10	0	-5	-1	0	-5	-1	94	96	92	92	93	91	100	100	99	93	93	91	60	43	55
		30	0	-5	-2	0	-6	-1	94	94	90	93	92	92	100	100	100	100	100	100	60	42	56
	
MD = 32	134/66	5	0	0	0	0	-1	1	96	96	96	88	88	88	100	100	100	91	91	91	92	91	91
RoM = 1.32		10	0	0	0	0	-1	0	96	96	96	92	92	91	100	100	100	100	100	100	92	91	91
		30	0	0	0	0	-1	0	96	96	95	94	94	93	100	100	100	100	100	100	92	91	91

Results of 10,000 simulations per scenario with a standard deviation equal to 40% of the control mean, for each combination of effect size (0.2, 0.5, and 0.8 standard deviation units), number of patients (14/6 and 134/66 experimental/control patients per trial), and number of trials (5, 10, and 30). The "% Bias" columns show the bias of each effect measure (MD, SMD, RoM) expressed as percentages of the expected values (negative sign denotes less than expected value), with and without heterogeneity. The "% coverage" columns show the percentage of cases that the true value falls within the 95% confidence interval of the simulated result, with and without heterogeneity. The "% statistical power" columns show the percentage of cases that the 95% confidence interval of the simulated result yields a significant treatment effect (i.e. excluding zero for MD and SMD, and one for RoM), with and without heterogeneity. The *I*^2^ column shows the degree of heterogeneity only for the scenarios in which heterogeneity was introduced. (For all the scenarios without heterogeneity, the ratio of Q/(k-1) was close to unity as expected, corresponding to *I*^2 ^= 0 [data not shown].)

Abbreviations for Table and Legend: contr – control, exp – experimental, *I*^2 ^– *I*^2 ^heterogeneity measure, k – number of trials in each meta-analysis, n – number of experimental or number of control patients per trial, Q – Cochran's Q statistic for heterogeneity, MD – mean difference, RoM – ratio of means, s – standard deviation units, SMD – standardized mean difference.

**Table 8:** Simulation Results (Normal Distribution, 1:2 Experimental to Control Group Sizes, Standard Deviation 40% of Control Mean Value).

			% Bias						% Coverage						% Statistical Power						*I*^2^(%)		
						
			*τ *= 0s			*τ *= 0.5s			*τ *= 0s			*τ *= 0.5s			*τ *= 0s			*τ *0.5s			*τ *0.5s		
							
Δ	n(exp/contr)	k	MD	SMD	RoM	MD	SMD	RoM	MD	SMD	RoM	MD	SMD	RoM	MD	SMD	RoM	MD	SMD	RoM	MD	SMD	RoM
SMD = 0.2	6/14	5	1	-3	1	1	-3	2	94	97	94	90	93	90	15	10	17	14	12	16	59	44	55
MD = 8		10	0	-4	1	2	-4	1	94	97	94	92	94	92	25	18	30	18	16	22	60	44	56
RoM = 1.08		30	0	-5	1	1	-5	1	93	97	93	94	94	93	59	53	69	36	35	44	60	44	56
	
	66/134	5	0	0	0	0	-1	1	96	96	96	88	88	87	77	77	79	20	20	21	92	91	92
		10	0	-1	0	0	-1	0	96	96	96	92	92	91	98	98	98	26	27	28	92	91	92
		30	0	0	0	0	-1	0	96	96	96	94	94	94	100	100	100	56	57	59	92	91	92

																							

SMD = 0.5	6/14	5	0	-3	1	1	-4	1	94	97	95	90	92	90	57	50	61	41	37	43	59	44	56
MD = 20		10	0	-4	1	1	-5	1	94	97	94	92	93	92	86	83	89	62	60	66	60	44	57
RoM = 1.2		30	0	-5	1	0	-6	1	93	96	94	94	94	94	100	100	100	96	97	98	60	43	57
	
	66/134	5	0	0	0	0	-1	1	96	96	96	88	88	88	100	100	100	61	61	61	92	91	92
		10	0	0	0	0	-1	0	96	96	96	92	92	91	100	100	100	84	85	86	92	91	92
		30	0	0	0	0	-1	0	96	96	96	94	94	94	100	100	100	100	100	100	92	91	92

																							

SMD = 0.8	6/14	5	0	-4	0	0	-4	1	94	97	95	90	92	90	91	89	93	72	69	74	59	43	56
MD = 32		10	0	-5	0	0	-5	1	94	96	95	92	93	92	100	100	100	93	93	94	60	43	57
RoM = 1.32		30	0	-5	0	0	-6	0	93	95	94	94	92	94	100	100	100	100	100	100	60	42	57
	
	66/134	5	0	0	0	0	-1	1	96	96	96	88	88	88	100	100	100	90	91	91	92	91	92
		10	0	0	0	0	-1	1	96	96	96	92	92	92	100	100	100	100	100	100	92	91	92
		30	0	0	0	0	-1	0	96	96	96	94	94	94	100	100	100	100	100	100	92	91	92

Results of 10,000 simulations per scenario with a standard deviation equal to 40% of the control mean, for each combination of effect size (0.2, 0.5, and 0.8 standard deviation units), number of patients (6/14 and 66/134 experimental/control patients per trial), and number of trials (5, 10, and 30). The "% Bias" columns show the bias of each effect measure (MD, SMD, RoM) expressed as percentages of the expected values (negative sign denotes less than expected value), with and without heterogeneity. The "% coverage" columns show the percentage of cases that the true value falls within the 95% confidence interval of the simulated result, with and without heterogeneity. The "% statistical power" columns show the percentage of cases that the 95% confidence interval of the simulated result yields a significant treatment effect (i.e. excluding zero for MD and SMD, and one for RoM), with and without heterogeneity. The *I*^2^ column shows the degree of heterogeneity only for the scenarios in which heterogeneity was introduced. (For all the scenarios without heterogeneity, the ratio of Q/(k-1) was close to unity as expected, corresponding to *I*^2 ^= 0 [data not shown].)

Abbreviations for Table and Legend: contr – control, exp – experimental, *I*^2 ^– *I*^2 ^heterogeneity measure, k – number of trials in each meta-analysis, n – number of experimental or number of control patients per trial, Q – Cochran's Q statistic for heterogeneity, MD – mean difference, RoM – ratio of means, s – standard deviation units, SMD – standardized mean difference.

##### RoM Bias Away from No Effect Due to Heterogeneity

The second RoM bias is a bias away from unity (or no effect) that occurs only in the scenarios with heterogeneity and is due to the effects of heterogeneity on the RoM. It is most apparent in the scenarios with heterogeneity with higher standard deviations (70% of the mean control value as shown in Table 5) when the number of patients per trial is 100, since in the 100-patient per trial scenarios the simultaneously present bias towards unity (or no effect) discussed above is less than 0.5%. (In the scenarios with heterogeneity and 10 patients per trial shown in Table 5, the overall bias is due to the combined effect of both the bias towards unity discussed in the previous section and the bias away from unity discussed in this section.) This bias away from unity ranges up to 1–2% in the scenarios with 100 patients per trial and occurs for the following reason. Heterogeneity is introduced in the simulations by shifting individual trial experimental and control mean values upwards or downwards. As in the scenarios presented, for RoM>1, in trials where both the experimental and control means are shifted upwards, the RoM value is decreased, and in trials when the means are both shifted downwards, the RoM value is increased. For upward and downward shifts of equal magnitude, the increase in the RoM for downward shifts is greater than the decrease in the RoM for upward shifts. This results in a pooled RoM value that is greater in the presence of heterogeneity and results in the bias away from unity. Table 5 demonstrates that this bias is higher for increasing effect sizes (i.e. larger ratios). Comparing the results where the standard deviation is higher (70% of the mean control value, Table 5) to those where the standard deviation is lower (e.g. 40% of the mean control value, Table 3) demonstrates that this bias increases with increasing standard deviations due to an increased range of individual trial RoM estimates.

### Coverage

The proportion of the scenarios for which the 95% confidence interval contains the true effect size is relatively similar among the three methods for most scenarios. The coverage is close to 95%, as expected, for the scenarios with no heterogeneity, but decreases when heterogeneity is introduced. The lowest coverage of 87–88% is equally low with all three methods and occurs when heterogeneity is present with 5 trials and 100 patients per trial arm. This low coverage occurs because with the degree of heterogeneity in these scenarios (*I*^2 ^= 92–93%) the mean values can be widely variable. With only 5 trials, the pooled value of these mean values can be far from the true value and due to the large number of patients per trial the confidence intervals for the individual trials are relatively narrow resulting in missed coverage of the true value. Increasing the number of patients to 1000 patients per trial arm still results in coverage rates between 87–88% (results not shown), because the degree of missed coverage is dominated by the degree of heterogeneity and the increase in *I*^2 ^from 92–93% in the scenarios with 100 patients per trial arm to *I*^2 ^= 99% for the scenarios with 1000 patients per trial arm is relatively small.

### Statistical Power to Detect a Significant Treatment Effect

As expected, statistical power (the proportion of scenarios yielding a significant treatment effect) increases with increasing effect size, number of patients, and number of trials, and decreases with more heterogeneity. Power also decreases with imbalanced patient allocation between groups (Tables 7 and 8) because confidence intervals are wider compared to balanced allocation scenarios. Statistical power is similar among the three methods in most scenarios. In scenarios where SMD or RoM are biased towards no effect, the power decreases compared to MD, and in the scenarios where RoM is biased away from unity, power increases compared to MD. Overall, the effect of these biases is small so that the proportion of scenarios yielding significant treatment effects is within 5 percentage points for almost all scenarios.

### Heterogeneity

For the scenarios with heterogeneity, *I*^2 ^is around 55–60% and greater than 90% for scenarios with n = 10 and n = 100 patients per trial, respectively, close to the expected values. In scenarios where SMD and RoM are biased, *I*^2 ^is lower compared to MD, which is relatively free of bias (for example, scenarios with 10 patients per trial in Tables 3, 4, 5 [SMD] or Table 5 [RoM]). This occurs because bias decreases the weighting of values greatly deviating from no effect (zero for SMD and one for RoM), decreasing *I*^2^. In the scenarios exhibiting less bias, *I*^2 ^among all methods is similar (for example, scenarios with 100 patients per trial in Tables 3, 4, 5).

## Discussion

This study examines the use of a new effect measure for meta-analysis of continuous outcomes that we call the ratio of means (RoM). In this method, the ratio of the mean value in the experimental group to that of the control group is calculated. The natural logarithm-transformed delta method approximated to first order terms provides a straightforward equation estimating the variance of the RoM for each study. Using this formulation, we performed simulations to compare the performance of RoM to traditionally used difference of means methods, MD and SMD.

Each method performed well within the simulated parameters with low bias and high coverage, even in scenarios with moderate or high heterogeneity. The methods had similar statistical power to detect significant treatment effects. SMD exhibited some bias towards zero or no effect, especially with smaller studies, as previously described [5,6], whereas MD was relatively bias-free. RoM gave acceptable results, with bias usually less than 2–3%.

As discussed earlier, SMD and RoM, unlike MD, allow pooling of studies expressed in different units and allow comparisons regarding relative effect sizes across different interventions. However, interpreting the results of a meta-analysis that uses SMD to determine the expected treatment effect in a specific patient population requires knowledge of the pooled standard deviation. This information is frequently unknown to clinicians. In contrast, interpretation of the results of a meta-analysis that uses RoM does not require knowledge of the pooled standard deviation and may permit clinicians to more readily estimate treatment effects for their patients. Moreover, RoM provides a result similar in form to a risk ratio, a binary effect measure preferred by clinicians [16]. Thus, overall RoM may be easier for clinicians to interpret.

One limitation of RoM is that the mean values of the intervention and control groups must both be positive or negative, since the logarithm of a negative ratio is undefined. All simulations assumed positive mean values in both groups. This limitation may be less important for biological variables since these generally have positive values. Another related limitation inherent to ratio methods occurs for a normally distributed control variable with a very broad distribution (i.e. a significant proportion of expected negative values) or for a control variable with only positive values but a distribution heavily skewed towards zero. In both such distributions, a high proportion of the control mean values will be very small. These small values in the denominator of the RoM can result in a high proportion of exceedingly large ratios. This could generate results biased to higher values.

In addition to statistical properties, the choice between a difference or a ratio method for a specific situation should be determined by the biological effect of the treatment as either additive or relative for different control group values. Unfortunately, this information is frequently not known in advance. For binary outcomes, empirical comparisons between difference methods (risk difference) and ratio methods (risk ratio and odds ratio) using published meta-analyses have shown that the risk difference exhibits less consistency compared to ratio methods, resulting in increased heterogeneity [17,18]. This suggests that for binary outcomes, relative differences are more preserved than absolute differences as baseline risk varies. It is unclear whether this is also the case for continuous outcomes, but such an empirical comparison between difference and ratio methods can be performed using our description of RoM.

## Conclusion

The results of our meta-analytic simulation studies suggest that the RoM method compares favorably to MD and SMD in terms of bias, coverage, and statistical power. Similar to binary outcome analysis for which both ratio and difference methods are available, this straightforward method provides researchers the option of using a ratio method in addition to difference methods for analyzing continuous outcomes.

## Abbreviations

CI – confidence interval; FE – fixed effects; IV – inverse variance; i – counter ranging from 1 to the number of trials in each meta-analysis (k); *I*^2 ^– *I*^2 ^heterogeneity measure; k – number of trials in each meta-analysis; MD – mean difference; mean_contr _– mean value in the control group; mean_exp _– mean value in the experimental group; n – number of experimental or number of control patients per trial; n_contr _– number of control patients per trial; n_exp _– number of experimental patients per trial; N – total number of patients per trial (N = n_contr _+ n_exp_); Q – Cochran's Q statistic for heterogeneity; RE – random effects; s – standard deviation; s^2 ^– sampling variance of the effect measure; SMD – standardized mean difference; SD – standard deviation; sd_contr _– standard deviation in the control group; sd_exp _– standard deviation in the experimental group; sd_pool _– pooled standard deviation of the control and experimental groups; *τ*^2 ^– variance due to heterogeneity; Var – variance; w_i _– weighting of study i; w_i _* – weighting of study i incorporating the variance due to heterogeneity; Θ_i _– MD, SMD, or RoM effect measure estimate for study i.

## Competing interests

The authors declare that they have no competing interests.

## Authors' contributions

JOF was involved with the conception and design of the study, acquisition, analysis and interpretation of data and drafted the manuscript. NKJA was involved with the conception and design of the study, interpretation of data and critical revision of the manuscript for important intellectual content. JB was involved in the conception and design of the study, interpretation of data and critical revision of the manuscript for important intellectual content. All authors read and approved the final version of the manuscript.

## Appendix

This appendix briefly reviews the inverse-variance weighted fixed and random effects models and the determination of the point estimate and variance for the continuous outcome measures, MD and SMD. The derivation of the point estimate and variance for RoM is described in the main text.

### The inverse-variance weighted fixed and random effects models

In fixed effects meta-analysis, the individual studies' treatment effect measures are assumed to be distributed around the same value for each study. An estimate of this effect measure is obtained by taking a weighted average of individual studies' effect measures, weighting each study by the inverse of the variance of the effect measure used:

$$\begin{array}{cc} \Theta_{\text{IV}(\text{FE})}=\frac{\Sigma_{\text{i}=1,\text{k}}{\text{w}}_{\text{i}}\times \Theta_{\text{i}}}{\Sigma_{\text{i}=1,\text{k}}{\text{w}}_{\text{i}}} & \text{with variance }(\Theta_{\text{IV}(\text{FE})})=1/\Sigma_{\text{i}=1,\text{k}}{\text{w}}_{\text{i}} \end{array}$$

where Θ_IV(FE) _is the inverse-variance weighted fixed effects pooled effect estimate for k total studies, Θ_i _is the effect measure estimate for study i, and weighting w_i _= 1/variance(Θ_i_).

In random effects meta-analysis, the individual studies' effect measures are assumed to vary around an overall average treatment effect. An estimate of the variance of this distribution of treatment effects, also known as between-study heterogeneity, *τ*^2^, is incorporated into the weights [13] to produce a summary estimate

$$\begin{array}{cc} \Theta_{\text{IV}(\text{RE})}=\frac{\Sigma_{\text{i}=1,\text{k}}{\text{w}}_{\text{i}}^{*}\times \Theta_{\text{i}}}{\Sigma_{\text{i}=1,\text{k}}{\text{w}}_{\text{i}}^{*}} & \text{with variance }(\Theta_{\text{IV}(\text{RE})})=1/\Sigma_{\text{i}=1,\text{k}}{\text{w}}_{\text{i}}^{*} \end{array}$$

where w_i_* = 1/(w_i_^-1 ^+ *τ*^2^). One estimate of *τ*^2 ^uses the Q statistic:

Q = Σ_i = 1, k _w_i _× (Θ_i _- Θ_IV(FE)_)^2^

which has a *χ*^2 ^distribution with k-1 degrees of freedom when *τ*^2 ^= 0. An estimate of *τ*^2 ^follows:

$$\begin{array}{ll} \tau^{2}=\frac{\text{Q}-(\text{k}-1)}{\Sigma_{\text{i}=1,\text{k}}{\text{w}}_{\text{i}}-\frac{\Sigma_{\text{i}=1,\text{k}}{\left({\text{w}}_{\text{i}}\right)}^{2}}{\Sigma_{\text{i}=1,\text{k}}{\text{w}}_{\text{i}}}} & \text{if Q}\geq \text{k}-1,\text{ and}\\ \tau^{2}=0 & \text{if Q}<\text{k}-1 \end{array}$$

When there is no between-trial heterogeneity (*τ*^2 ^= 0), the Q-statistic has the expected value of k-1, and the ratio Q/(k-1) [12] has an expected value of unity. Under these circumstances the random effects model is equivalent to the fixed effects model. In situations with heterogeneity (*τ*^2 ^> 0), Q/(k-1)> 1, and the proportion of variation in study-level estimates of treatment effect due to between-study heterogeneity can be expressed using the *I*^2 ^measure expressed as a percentage. *I*^2 ^can be expressed in terms of Q and k-1, where *I*^2 ^= [Q/(k-1) - 1]/[Q/(k-1)] which simplifies to (Q-(k-1))/Q [12]. *I*^2 ^can also be expressed as *τ*^2^/(*τ*^2 ^+ s^2^), where s^2 ^is the variance of the effect measure, and s^2 ^= Σ_i = 1, k _w_i _(k-1)/[Σ_i = 1, k _w_i_)^2 ^- Σ_i = 1, k _w_i_^2^] [12]. When the variance (and thus weighting) of each trial is identical, as is the case with all the simulated scenarios in this study, then the variance of the effect measure, or s^2^, reduces to the variance of a single trial.

Thus, to carry out a random effects meta-analysis requires calculating the effect measure and its variance for each study to be combined. First the fixed effects pooled effect measure is calculated, which is then used to estimate Q and *τ*^2^, and finally *τ*^2 ^is used to estimate the random effects pooled effect measure and its variance.

### The Mean Difference Effect Measure

Using the measured values, the mean difference effect measure for each study (MD_i_) is estimated as:

MD_i _= *mean*_*exp *_- *mean*_*contr*_

with estimated variance,

*Var *(MD_i_) = *Var *(*mean*_*exp*_) + *Var *(*mean*_*contr*_) = (*sd*_*exp*_/√*n*_*exp*_)^2 ^+ (*sd*_*contr*_/√*n*_*contr*_)^2^

where the subscripts "*exp*" and "*contr*" refer to the experimental and control groups, respectively, *mean *to the mean value, *sd *to the standard deviation, and *n *to the number of patients in each group. The individual effect measures and their variances are combined as described previously. All studies need to be reported in identical units for mean difference to be used as the effect measure.

### The Standardized Mean Difference Effect Measure

When the outcome is not measured in identical units across studies, one can use the standardized mean difference for each study (SMD_i_), in which the difference in the means is divided by the pooled standard deviation. The estimated value of SMD_i _is often multiplied by a correction factor to correct for bias away from zero (towards larger effect sizes) when the number of patients in each group is small [5], as follows:

$${\text{SMD}}_{\text{i}}=\frac{\left(mean_{exp}-mean_{contr}\right)}{sd_{pool}}\times [1-\frac{3}{\left(4N-9\right)}]$$

with estimated variance,

$$Var({\text{SMD}}_{\text{i}})=\frac{\text{N}}{n_{exp}n_{contr}}+\frac{{\text{SMD}}_{\text{i}}^{2}}{2(\text{N}-3.94)}$$

where

$$\begin{array}{l} N=n_{exp}+n_{contr}\text{ and}\\ sd_{pool}=\surd \frac{\left[(n_{exp}-1)sd_{exp}^{2}+(n_{contr}-1)sd_{contr}^{2}-1\right]}{N-2} \end{array}$$

The individual effect measures and their variances are combined as described previously. As SMD_i_ assumes more extreme positive or negative values deviating from zero, *Var*(SMD_i_) increases, resulting in a smaller weighting for such trials. This means that in general SMD is biased towards zero or no effect [5,6]. This bias towards zero is independent of the number of studies in the meta-analysis, and decreases for larger N. Using a random effects model instead of a fixed effects model can reduce this bias because the between-study variance, estimated by *τ*^2^, tends to equalize the study weights. However, this advantage is offset by a lower Q (used to estimate *τ*^2^), which depends on the inverse of the variance for each study and therefore is also biased towards lower values. Alternate weighting methods have been proposed to address this bias [5,6].

## Pre-publication history

The pre-publication history for this paper can be accessed here:


